# A Bilateral Carotid Web Presenting as Recurrent Transient Ischemic Attacks With Negative Initial Stroke Imaging: A Case Report

**DOI:** 10.7759/cureus.110063

**Published:** 2026-06-01

**Authors:** Daniel Balderrama

**Affiliations:** 1 Internal Medicine, Hospital Metropolitano, San Nicolás de los Garza, MEX

**Keywords:** carotid stent, carotid web, coronary artery angiography, coronary fibromuscular dysplasia, cryptogenic strokes, neurovascular surgery, recurrent ischemic stroke, transient ischemic attack (tia)

## Abstract

Carotid webs (CWs) are an increasingly recognized cause of ischemic stroke and transient ischemic attacks (TIAs), particularly in patients without an alternative clearly identified etiology, although they likely remain underdiagnosed because of their subtle radiographic appearance and the absence of significant carotid stenosis. The lesion is considered an intimal variant of fibromuscular dysplasia that may promote local blood stasis, thrombus formation, and distal cerebral embolization.

We present the case of a 59-year-old woman with recurrent transient neurological deficits and initially negative stroke imaging who was ultimately diagnosed with a bilateral CW. Computed tomography angiography demonstrated bilateral thin linear filling defects at the carotid bulbs, more prominent on the left side, consistent with a CW. Magnetic resonance imaging performed using a stroke protocol showed no evidence of acute ischemic or hemorrhagic stroke. Electroencephalography was normal, and no definitive cardioembolic source was identified. Subsequent digital subtraction angiography confirmed abnormal flow dynamics involving both carotid bulbs. Balloon occlusion testing demonstrated insufficient collateral circulation, supporting the hemodynamic significance of the lesion. Given the recurrent ischemic symptoms and angiographic findings, the patient underwent endovascular carotid reconstruction with angioplasty and placement of a left internal carotid artery stent. Post-procedural angiography demonstrated improved carotid flow dynamics without complications. The patient remained neurologically stable during follow-up without recurrent cerebrovascular events.

This case highlights the importance of considering CWs in patients with recurrent cryptogenic TIAs or stroke-like symptoms despite negative initial neuroimaging. Early recognition through dedicated vascular imaging may facilitate timely intervention and potentially reduce recurrent cerebrovascular events.

## Introduction

Carotid webs (CWs) are increasingly recognized as an underdiagnosed cause of ischemic stroke and transient ischemic attacks (TIAs), particularly in patients with otherwise unexplained cerebrovascular events without significant atherosclerotic disease or another clearly identifiable embolic source [1,2]. Histopathologically, a CW is considered an intimal variant of fibromuscular dysplasia characterized by a thin shelf-like intraluminal projection that typically arises from the posterior wall of the carotid bulb [1-3].

Although a CW may not produce hemodynamically significant stenosis, it can alter local blood flow dynamics, promoting blood stasis, thrombus formation, and distal cerebral embolization [1,2]. Previous studies have demonstrated a significant association between symptomatic CWs and recurrent ischemic events, particularly in patients with cryptogenic stroke [4,5]. Computed tomography angiography (CTA) with multiplanar reconstruction has emerged as one of the most useful diagnostic modalities for lesion identification [6].

Recent evidence suggests that a symptomatic CW is associated with a substantially increased risk of recurrent ischemic stroke despite antithrombotic therapy alone [7,8]. Furthermore, systematic reviews and meta-analyses have reported lower recurrence rates in patients undergoing carotid intervention, including carotid artery stenting and carotid endarterectomy, compared with conservative medical management [9-14]. Despite growing recognition of this entity, optimal management strategies remain incompletely defined, and current guidelines acknowledge CWs as a potential cause of cryptogenic stroke while emphasizing the need for further evidence regarding treatment selection [15].

We present the case of a patient with a bilateral CW associated with recurrent transient neurological deficits and initially negative stroke imaging, highlighting the importance of early vascular imaging and recognition of this underdiagnosed cause of cryptogenic cerebrovascular events.

## Case presentation

A 59-year-old woman with recurrent transient neurological episodes presented to the emergency department with acute right-sided neurological symptoms. Since 2014, she had experienced multiple transient episodes characterized by incoherent speech, right hemiparesis, and facial asymmetry with spontaneous resolution after several minutes. Her medical history was significant for asthma and an unspecified cardiac arrhythmia. She was receiving aspirin 100 mg daily and denied previous anticoagulation therapy. A summary of the patient’s clinical course, diagnostic evaluation, and therapeutic interventions is presented in Table 1.

**Table 1:** Clinical timeline, diagnostic evaluation, and therapeutic interventions. MRI: magnetic resonance imaging; TIA: transient ischemic attack.

Timeline/Event	Clinical Findings and Interventions
2014	First transient neurological episodes characterized by incoherent speech, right hemiparesis, and facial asymmetry with spontaneous resolution
Prior medical history	Asthma, recurrent transient ischemic attacks, and unspecified cardiac arrhythmia
Chronic treatment before admission	Aspirin 100 mg daily
24 hours before admission	Sudden right upper extremity pain followed by severe right temporal oppressive headache rated 9/10 in intensity
Hours before admission	Development of transient right upper extremity weakness, right lower extremity pain, and paresthesias involving the right hemibody
Emergency department evaluation	Stroke protocol activated
Initial neurological examination	Preserved consciousness and orientation without persistent focal neurological deficits
Electrocardiography	Ventricular ectopy and possible atrial rhythm abnormalities
Brain MRI stroke protocol	No evidence of acute ischemic or hemorrhagic stroke; mild chronic microangiopathic changes (Fazekas grade 1)
Electroencephalography	Normal study without epileptiform activity
Computed tomography angiography	Bilateral carotid web involving the carotid bulbs and proximal internal carotid arteries, more prominent on the left side
Diagnostic cerebral angiography	Abnormal flow dynamics at both carotid bulbs consistent with a bilateral carotid web
Balloon occlusion testing	Inadequate collateral circulation through the right A1 segment
Endovascular treatment	Left carotid angioplasty with tapered XACT carotid stent placement
Post-procedural angiography	Improved carotid flow dynamics following angioplasty and carotid stent placement
Follow-up	Three-month follow-up. No recurrent neurological deficits, ischemic stroke, or additional cerebrovascular events

Twenty-four hours before admission, she developed sudden right upper extremity pain followed by severe right temporal oppressive headache rated 9/10 in intensity. Hours later, she experienced right upper extremity weakness, right lower extremity pain, and paresthesias involving the right hemibody, prompting evaluation in the emergency department, where a stroke protocol was activated.

On admission, vital signs showed blood pressure of 124/77 mmHg, heart rate of 58 beats/minute, respiratory rate of 22 breaths/minute, oxygen saturation of 100% on room air, and temperature of 36.6°C. Initial neurological examination demonstrated preserved consciousness and orientation without persistent focal neurological deficits. Motor strength was 5/5 in all extremities, cranial nerves were intact, and no sensory deficits were identified at the time of evaluation.

Initial laboratory evaluation included complete blood count, comprehensive metabolic panel, coagulation profile, venous blood gas analysis, and electrolyte assessment (Table 2). Venous blood gas analysis demonstrated pH 7.45, pCO2 33 mmHg, bicarbonate 22.3 mmol/L, lactate 1.6 mmol/L, and oxygen saturation of 88%. Complete blood count revealed hemoglobin 14.3 g/dL, leukocytes 4.63 ×10³/µL, and platelets 163 ×10³/µL. The metabolic panel demonstrated glucose 113 mg/dL, creatinine 0.7 mg/dL, sodium 138.7 mmol/L, potassium 3.95 mmol/L, and chloride 105.3 mmol/L. Liver function tests, coagulation profile, and remaining laboratory parameters were within normal limits (Table 2). Electrocardiography demonstrated ventricular ectopy and possible atrial rhythm abnormalities without definitive evidence of sustained atrial fibrillation (Figure 1). Subsequent cardiologic evaluation did not confirm sustained atrial fibrillation. A multidisciplinary neurovascular evaluation was subsequently performed.

**Figure 1:**
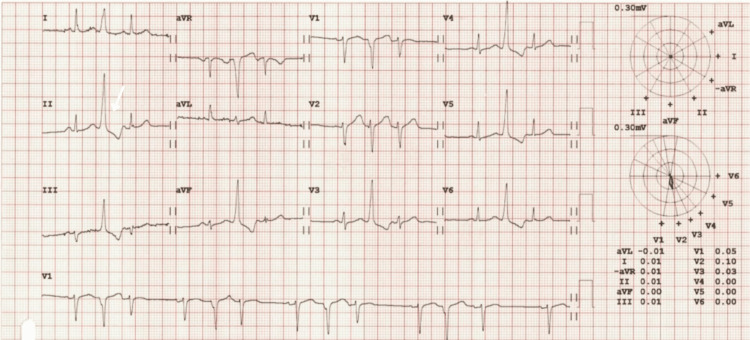
Electrocardiogram obtained during initial evaluation. Electrocardiography demonstrated ventricular ectopy and nonspecific atrial rhythm abnormalities without definitive evidence of sustained atrial fibrillation. The arrow indicates a premature ventricular complex. Paper speed: 25 mm/s; calibration: 10 mm/mV.

**Table 2:** Initial laboratory findings on admission.

Laboratory Test	Result	Reference range
Venous pH	7.45	7.31–7.41
pCO₂	33 mmHg	41–51 mmHg
Oxygen saturation	88%	60–80%
Bicarbonate	22.3 mmol/L	22–26 mmol/L
Lactate	1.6 mmol/L	0.5–2.0 mmol/L
Ionized calcium	1.17 mmol/L	1.12–1.32 mmol/L
Anion gap	6	3–11
Hemoglobin	14.3 g/dL	12.0–16.0 g/dL
Hematocrit	41.7%	36–46%
Erythrocytes	4.41 ×10⁶/µL	4.0–5.2 ×10⁶/µL
Mean corpuscular volume	94.6 fL	80–100 fL
Mean corpuscular hemoglobin	32.4 pg	27–33 pg
Mean corpuscular hemoglobin concentration	34.3 g/dL	32–36 g/dL
Red cell distribution width	12.3%	11.5–14.5%
Leukocytes	4.63 ×10³/µL	4.0–10.0 ×10³/µL
Neutrophils	2.3 ×10³/µL	1.8–7.0 ×10³/µL
Lymphocytes	1.86 ×10³/µL	1.0–4.0 ×10³/µL
Eosinophils	0.18 ×10³/µL	0.0–0.5 ×10³/µL
Platelets	163 ×10³/µL	150–450 ×10³/µL
Glucose	113 mg/dL	70–100 mg/dL
Creatinine	0.7 mg/dL	0.5–1.1 mg/dL
Urea	27 mg/dL	15–40 mg/dL
Blood urea nitrogen	13 mg/dL	7–20 mg/dL
Uric acid	4.8 mg/dL	2.4–6.0 mg/dL
Total cholesterol	122 mg/dL	<200 mg/dL
Triglycerides	109 mg/dL	<150 mg/dL
Total bilirubin	0.51 mg/dL	0.2–1.2 mg/dL
Direct bilirubin	0.31 mg/dL	0.0–0.3 mg/dL
Indirect bilirubin	0.20 mg/dL	0.2–0.8 mg/dL
Aspartate aminotransferase	30 U/L	10–40 U/L
Alanine aminotransferase	31 U/L	7–56 U/L
Total protein	6.7 g/dL	6.0–8.3 g/dL
Albumin	4.0 g/dL	3.5–5.0 g/dL
Globulin	2.2 g/dL	2.0–3.5 g/dL
Calcium	9.1 mg/dL	8.5–10.5 mg/dL
Phosphorus	2.3 mg/dL	2.5–4.5 mg/dL
Lactate dehydrogenase	167 U/L	140–280 U/L
Alkaline phosphatase	56 U/L	44–147 U/L
Serum iron	79 µg/dL	50–170 µg/dL
Magnesium	1.38 mg/dL	1.7–2.2 mg/dL
Sodium	138.7 mmol/L	135–145 mmol/L
Potassium	3.95 mmol/L	3.5–5.1 mmol/L
Chloride	105.3 mmol/L	98–107 mmol/L
Prothrombin time	14 sec	11–14 sec
Activated partial thromboplastin time	29.7 sec	25–35 sec
International normalized ratio (INR)	1.1	0.8–1.2

Magnetic resonance imaging (MRI) using a stroke protocol, including diffusion-weighted imaging, fluid-attenuated inversion recovery (FLAIR), susceptibility-weighted imaging, and 3D time-of-flight sequences, demonstrated no evidence of acute ischemic or hemorrhagic stroke. No diffusion restriction, vascular thrombus, or abnormal vascular prominence was identified. Mild punctate subcortical white matter hyperintensities consistent with chronic microangiopathic changes (Fazekas grade 1) and mild generalized cortical-subcortical atrophy (GCA grade 1) were observed. Electroencephalography demonstrated normal background activity without epileptiform discharges.

Subsequent CTA of the head and neck demonstrated normal caliber and course of the carotid and vertebral arteries without significant stenosis. However, thin shelf-like intraluminal filling defects involving the carotid bulbs and proximal internal carotid arteries, more prominent on the left side, were identified and considered highly suggestive of a bilateral CW (Figure 2). No aneurysm, vascular malformation, or acute intracranial hemorrhage was observed.

**Figure 2:**
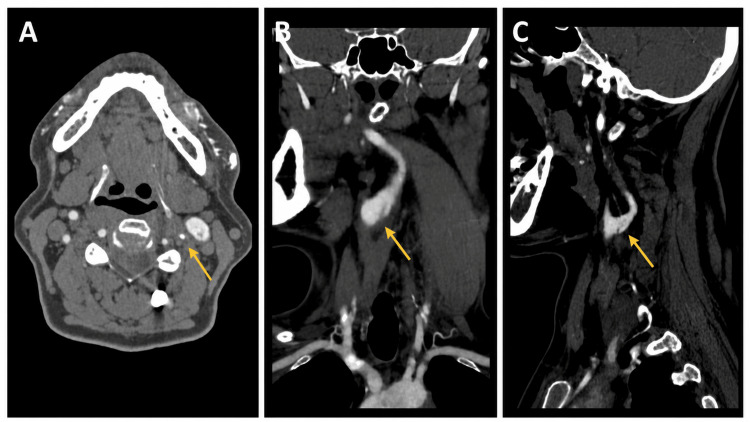
Multiplanar computed tomography angiography demonstrating the left carotid web. (A) Axial computed tomography angiography demonstrating a subtle intraluminal filling defect at the left carotid bulb (arrow). (B) Coronal computed tomography angiography showing a thin shelf-like projection arising from the posterior wall of the proximal internal carotid artery (arrow). (C) Sagittal computed tomography angiography demonstrating the characteristic shelf-like intraluminal projection consistent with a carotid web (arrow).

Given the recurrent transient neurological symptoms and vascular imaging findings, the patient underwent diagnostic cerebral angiography. Angiographic evaluation confirmed abnormal flow dynamics at both carotid bulbs, more significant on the left side, consistent with a bilateral CW previously identified on CTA. Balloon occlusion testing demonstrated insufficient collateral circulation, with only minimal flow through the right A1 segment, precluding safe vessel occlusion.

Due to the recurrent ischemic symptoms and angiographic findings, the patient subsequently underwent endovascular carotid reconstruction of the left internal carotid artery under general anesthesia. Angioplasty with balloon dilation and placement of a tapered XACT carotid stent was successfully performed. Post-procedural angiography demonstrated improved carotid flow dynamics following angioplasty and carotid stent placement (Figure 3). No intraoperative or immediate postoperative complications occurred.

**Figure 3:**
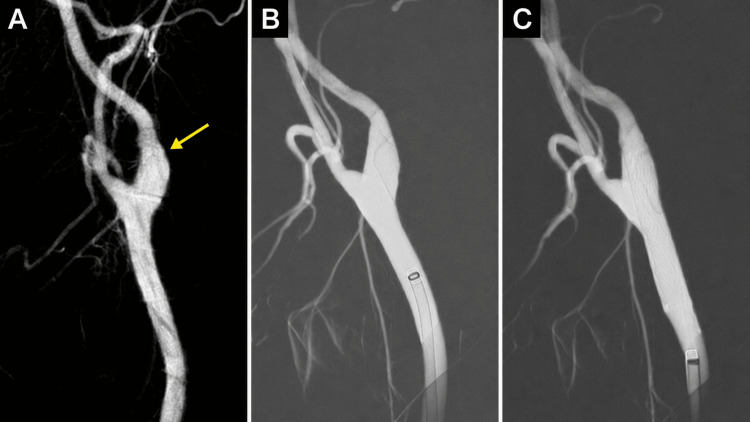
Digital subtraction angiography and endovascular treatment of the left carotid web. (A) Pre-intervention angiography demonstrating abnormal flow dynamics at the left carotid bulb consistent with a carotid web (arrow). (B) Deployment of a tapered carotid stent during endovascular reconstruction. (C) Final angiographic result after angioplasty and stent placement demonstrating improved carotid flow dynamics.

During the three-month follow-up, the patient remained neurologically stable without recurrent transient neurological deficits, ischemic stroke, or additional cerebrovascular events.

## Discussion

CWs have emerged as an increasingly recognized cause of embolic stroke without an alternative clearly identified etiology, particularly in patients without significant carotid stenosis or another clearly identifiable embolic source [1,2]. Histopathologically, CWs are considered intimal variants of fibromuscular dysplasia characterized by focal intimal hyperplasia forming a shelf-like intraluminal projection, most commonly arising from the posterior wall of the carotid bulb [1-3]. Although frequently non-stenotic, these lesions may alter local blood flow dynamics, resulting in blood stasis, thrombus formation, and distal cerebral embolization [1,2].

The present case illustrates several clinically relevant features associated with a symptomatic CW. The patient experienced recurrent transient neurological deficits over several years despite the absence of significant carotid stenosis or acute ischemic lesions on neuroimaging. Previous studies have shown that CWs may remain undetected during the evaluation of cryptogenic cerebrovascular events because the lesion often does not produce significant luminal narrowing and may be misinterpreted as minimal atherosclerotic plaque or vascular artifact [1,2]. In this patient, CTA with multiplanar reconstruction demonstrated bilateral thin linear filling defects arising from the carotid bulbs, findings highly suggestive of a bilateral CW, later confirmed by digital subtraction angiography.

Bilateral involvement has been described in previous studies and may represent a marker of increased vascular susceptibility [5,6]. In the present case, angiographic evaluation demonstrated abnormal flow dynamics affecting both carotid bulbs, with more pronounced involvement on the left side, correlating with the patient’s recurrent neurological symptoms. In addition, balloon occlusion testing demonstrated insufficient collateral circulation, further supporting the potential hemodynamic relevance of the lesion.

Another important aspect of this case is the absence of acute infarction on MRI despite clinically significant transient focal deficits. Previous reports have emphasized that CWs may present with recurrent TIAs or embolic events without persistent ischemic lesions, particularly during transient or early presentations [4,5]. This diagnostic discordance may delay recognition of the underlying vascular abnormality and contribute to recurrent ischemic episodes. In our patient, stroke protocol MRI showed no evidence of acute ischemia or hemorrhage, while electroencephalography was normal, and no definitive cardioembolic source was identified despite initial rhythm abnormalities on electrocardiography. Recognition of these subtle vascular abnormalities may be particularly challenging in patients with transient symptoms and initially unrevealing neuroimaging studies.

Recent evidence suggests that a symptomatic CW is associated with a substantial risk of recurrent ischemic stroke despite antithrombotic therapy alone [7,8]. Delayed recognition of CWs has also been associated with significantly increased recurrence risk, highlighting the importance of early vascular imaging and diagnosis [8]. Multiple systematic reviews and meta-analyses have demonstrated lower recurrence rates following carotid intervention compared with conservative medical therapy [9-14]. Recurrent ischemic events have been consistently reported in medically managed patients, whereas markedly lower recurrence rates have been observed following carotid revascularization with carotid artery stenting or carotid endarterectomy [9-14].

In the present case, diagnostic angiography demonstrated abnormal carotid flow dynamics and insufficient collateral circulation during balloon occlusion testing, supporting the potential hemodynamic relevance of the lesion. The patient subsequently underwent endovascular carotid reconstruction with angioplasty and carotid stent placement, resulting in improved angiographic flow and reduction of delayed contrast stasis within the carotid bulb. No perioperative complications occurred, and the patient remained neurologically stable during follow-up.

Current AHA/ASA guidelines recognize CWs as a potential cause of cryptogenic stroke but acknowledge that evidence regarding optimal treatment selection remains limited because randomized controlled trials are lacking [15]. Nevertheless, growing observational evidence supports consideration of carotid intervention in carefully selected symptomatic patients, particularly those with recurrent ischemic events despite medical therapy [9-15].

The clinical relevance of this case lies in the diagnostic challenge posed by recurrent transient neurological deficits despite negative initial stroke imaging and the eventual identification of the bilateral CW through dedicated vascular imaging and angiographic evaluation. Increased awareness of this entity may facilitate earlier diagnosis and potentially reduce recurrent cerebrovascular events in selected patients.

This report has several limitations inherent to single-case studies. Histopathological confirmation of the CW was not available because the patient underwent endovascular treatment rather than surgical resection. In addition, long-term follow-up remains limited, restricting assessment of long-term recurrence prevention after intervention. Despite these limitations, the present case provides clinically relevant radiographic and angiographic correlation supporting the association between the CW and recurrent transient ischemic events.

Clinicians should maintain a high index of suspicion for CWs in patients with recurrent cryptogenic TIAs or embolic stroke-like episodes despite negative initial neuroimaging, particularly when conventional etiologies are not identified. Early vascular imaging with CTA and timely recognition may facilitate definitive treatment and potentially reduce recurrent cerebrovascular events.

## Conclusions

CWs are an increasingly recognized and likely underdiagnosed cause of cryptogenic ischemic stroke and TIAs, particularly in patients without significant atherosclerotic disease or another clearly identifiable embolic source. This case highlights the diagnostic challenges associated with recurrent transient neurological deficits in the setting of initially negative stroke imaging and emphasizes the importance of dedicated vascular imaging for identifying subtle carotid abnormalities.

In this patient, a bilateral CW was associated with abnormal angiographic flow dynamics and insufficient collateral circulation, supporting the hemodynamic relevance of the lesion. Endovascular carotid reconstruction with angioplasty and stent placement resulted in improved carotid flow without procedural complications or recurrent neurological events during follow-up. Early recognition of CWs may facilitate timely diagnosis and intervention, potentially reducing the risk of recurrent cerebrovascular events in carefully selected symptomatic patients.

## References

[1] Mac Grory B, Emmer BJ, Roosendaal SD, Zagzag D, Yaghi S, Nossek E (2020). Carotid web: an occult mechanism of embolic stroke. J Neurol Neurosurg Psychiatry.

[2] Kim SJ, Nogueira RG, Haussen DC (2019). Current understanding and gaps in research of carotid webs in ischemic strokes: a review. JAMA Neurol.

[3] Wang LZ, Calvet D, Julia P (2023). Is carotid web an arterial wall dysplasia? A histological series. Cardiovasc Pathol.

[4] Mac Grory B, Nossek E, Reznik ME (2021). Ipsilateral internal carotid artery web and acute ischemic stroke: a cohort study, systematic review and meta-analysis. PLoS One.

[5] Haussen DC, Grossberg JA, Bouslama M (2017). Carotid web (intimal fibromuscular dysplasia) has high stroke recurrence risk and is amenable to stenting. Stroke.

[6] Zhu C, Li Z, Ju Y, Zhao X (2021). Detection of carotid webs by CT angiography, high-resolution MRI, and ultrasound. J Neuroimaging.

[7] Guglielmi V, Compagne KC, Sarrami AH (2021). Assessment of recurrent stroke risk in patients with a carotid web. JAMA Neurol.

[8] Poirette P, Chausson N, Papaxanthos J (2025). Delay in carotid web diagnosis remains common and associated with an increased risk of stroke recurrence. Stroke.

[9] Zhang AJ, Dhruv P, Choi P (2018). A systematic literature review of patients with carotid web and acute ischemic stroke. Stroke.

[10] Khan F, Rehman CA, Fletcher L (2025). Carotid revascularization versus medical management for ischemic stroke with ipsilateral carotid web: a systematic review and meta-analysis. Ann Neurol.

[11] Xenos D, Texakalidis P, Giannopoulos S (2026). Clinical characteristics and medical versus interventional management of carotid artery webs: a systematic review and meta-analysis. J Neurointerv Surg.

[12] Rosso M, Grin EA, Chen A (2026). Pathologically confirmed symptomatic carotid webs: histopathological features and long-term surgical outcomes. Neurosurgery.

[13] Brinster CJ, O'Leary J, Hayson A (2024). Symptomatic carotid webs require aggressive intervention. J Vasc Surg.

[14] Patel SD, Otite FO, Topiwala K (2022). Interventional compared with medical management of symptomatic carotid web: a systematic review. J Stroke Cerebrovasc Dis.

[15] Kleindorfer DO, Towfighi A, Chaturvedi S (2021). 2021 guideline for the prevention of stroke in patients with stroke and transient ischemic attack: a guideline from the American Heart Association/American Stroke Association. Stroke.

